# Effect of intraoperative autotransfusion use during liver transplantation for hepatocellular carcinoma on recurrence-free survival: comparative study with propensity score matching

**DOI:** 10.1093/bjsopen/zraf101

**Published:** 2025-09-09

**Authors:** Paul Boulard, Charlotte Maulat, Ana Cavillon, Fabien Robin, Frederica Dondero, Chady Salloum, Celia Turco, Flavy Breheret, Valérie Paradis, Chetana Lim, Bruno Heyd, Emmanuel Cuellar, Bertrand Suc, Daniel Azoulay, Isabelle Migueres, François Cauchy, Fabrice Muscari

**Affiliations:** Digestive Surgery and Transplantation Department, Toulouse University Hospital Centre, Toulouse, France; Digestive Surgery and Transplantation Department, Toulouse University Hospital Centre, Toulouse, France; Biostatistics and Health Data Science Unit, Claudius-Regaud Institute, IUCT-Oncopole, Toulouse, France; Digestive Surgery, Rennes University Hospital Centre, Rennes, France; Digestive Surgery, Beaujon University Hospital Centre, Clichy, France; Digestive Surgery, Créteil University Hospital Centre, Créteil, France; Digestive Surgery, Besançon University Hospital Centre, Besançon, France; Digestive Surgery, Rennes University Hospital Centre, Rennes, France; Digestive Surgery, Créteil University Hospital Centre, Créteil, France; Digestive Surgery, Créteil University Hospital Centre, Créteil, France; Digestive Surgery, Besançon University Hospital Centre, Besançon, France; Digestive Surgery and Transplantation Department, Toulouse University Hospital Centre, Toulouse, France; Digestive Surgery and Transplantation Department, Toulouse University Hospital Centre, Toulouse, France; Digestive Surgery, Créteil University Hospital Centre, Créteil, France; Anaesthesiology Department, Toulouse University Hospital Centre, Toulouse, France; Digestive Surgery, Beaujon University Hospital Centre, Clichy, France; Digestive Surgery and Transplantation Department, Toulouse University Hospital Centre, Toulouse, France

## Abstract

**Background:**

Intraoperative autotransfusion remains underutilized in high-risk haemorrhagic oncological procedures, particularly in liver transplantation for hepatocellular carcinoma. This is because of the theoretical risk of tumour cell reinfusion and dissemination, potentially leading to reduced recurrence-free survival. The aim of this study was to evaluate the impact of intraoperative autotransfusion on recurrence-free survival during liver transplantation for hepatocellular carcinoma.

**Methods:**

This was a retrospective study of patients receiving liver transplantation for hepatocellular carcinoma with or without intraoperative autotransfusion between 1 January 2011 and 1 January 2020 at five French hospitals, of which one used autotransfusion and four did not. Propensity score matching was used to match the cohorts with and without autotransfusion. The primary endpoint was 5-year recurrence-free survival.

**Results:**

Some 113 patients in the study cohort (autotransfusion) were compared with 441 patients in the control cohort. The median volume of autotransfused blood was 1500 ml. Median follow-up was 84.6 months. There was no significant difference in 5-year recurrence-free survival between the cohorts (69.7% in control cohort *versus* 66.3% in study cohort; *P* = 0.241). After matching patients based on oncological criteria, the difference remained non-significant, with a 5-year recurrence-free survival rate of 67.1% in the study cohort and 77.6% in the control cohort (*P* = 0.174).

**Conclusion:**

The use of autotransfusion during liver transplantation for hepatocellular carcinoma was not associated with recurrence-free survival.

## Teaser

Using propensity score matching, this retrospective study compared a cohort of patients undergoing liver transplantation for hepatocellular carcinoma with autotransfusion and a cohort without autotransfusion. The use of autotransfusion during liver transplantations for hepatocellular carcinoma did not influence recurrence-free survival. The use of autotransfusion made it possible to avoid the use of 5 units packed red blood cells per transplant without altering the oncological prognosis.

## Introduction

Liver transplantation (LT) for hepatocellular carcinoma (HCC) accounted for 18 000 cases between 1968 and 2016 in Europe^1^. This approach represents the best curative option for patients with HCC, as it treats both the tumour and the underlying cirrhosis—the main risk factor for HCC—with 10-year survival rates of 60–70%^2^. The recurrence rate varies between 10 and 15%^3^, with recurrence most often occurring within 2 years after LT^4^. These promising results have been achieved through the use of the Milan criteria since 1996^5^, and, more recently in France, by adopting the α-fetoprotein (AFP) score^6^, which has improved patient selection for LT in patients with HCC.

LT is a complex procedure with a high risk of bleeding, owing to both the surgical technique itself and the consequences of cirrhosis, including coagulation disorders and portal hypertension. Developments in surgical and anaesthetic techniques have led to a reduction in bleeding during LT. However, a large proportion (50–63%) of patients having a transplant receive allogeneic transfusions of packed red blood cells (RBCs)^7^, with a median of 4 units per patient^8^.

The risks of allogeneic blood transfusion are well known, including an increased risk of postoperative infections, higher morbidity and mortality rates, and longer hospital stay^9^. In oncological surgery, such transfusions have been associated with a decreased chance of long-term survival and increased recurrence risk^10,11^. The growing shortage of blood in blood banks, along with the cost of its use, have largely contributed to the rise in intraoperative autotransfusion in high-risk bleeding procedures, particularly in cardiac, vascular, and orthopaedic surgery^12^.

Intraoperative autotransfusion remains underutilized in high-risk haemorrhagic oncological procedures throughout the world^13^, because of the theoretical risk of tumour cell reinfusion and dissemination, as well as its potential association with reduced recurrence-free survival (RFS). However, no published study on autotransfusion in oncological surgery—regardless of the type of cancer (for example prostate^14^, colon^15^, HCC^16–27^)—has demonstrated any negative impact on recurrence and survival. The few available *in vitro* studies^28^ have shown that cancer cells were absent at the outlet port of the blood salvage and intraoperative autotransfusion system, particularly with the addition of an antileucocyte filter^29^. Yet, although not contraindicated in oncological surgery by the European Society of Anaesthesiology^30^, intraoperative autotransfusion remains underused, if used at all, particularly in LT for HCC. Notably, a recent meta-analysis^7^ found no detrimental effect on recurrence and survival with use of it during LT for HCC.

The primary aim of this study was to investigate the outcomes of patients receiving LT for HCC with the use of intraoperative autotransfusion, and to compare their RFS with that of a control cohort of patients receiving LT for HCC without intraoperative autotransfusion in four high-volume French centres. Additionally, prognostic factors for overall survival (OS) and RFS were investigated.

## Methods

The autotransfusion cohort included all patients who underwent LT for HCC with intraoperative autotransfusion between 1 January 2011 and 1 January 2020 at Toulouse University Hospital Centre. In the Toulouse centre, intraoperative autotransfusion is used in all LTs, for all indications, including HCC. The control cohort, used for comparison of RFS, comprised all patients who underwent LT for HCC without intraoperative autotransfusion between 1 March 2013 and 1 January 2018 at Besançon, Beaujon, Créteil, and Rennes University Hospitals. These centres do not use intraoperative autotransfusion for cancer transplants. For nine patients in Toulouse, the quantity of blood received by the autotransfusion machine did not allow retransfusion, so they were included in the control cohort.

### Inclusion and exclusion criteria

Inclusion criteria were registration on the list and completion of LT for HCC developed on cirrhotic liver, and age ≥ 18 years at the time of transplantation. Patients whose HCC was found only on histological analysis of the surgical specimen were excluded, as were those who had no HCC detected on definitive histological analysis.

### Data collection

Data were originated from electronic patient files for each centre and captured retrospectively. Fewer data were available in the multicentre control cohort than in the single-centre autotransfusion cohort, particularly regarding intraoperative and postoperative variables. All the data available for each cohort are reported in *Table S1*.

### Study endpoints

The primary endpoint was 5-year RFS and 5-year OS was a secondary endpoint.

### Registration on LT list and transplant allocation

Patients were registered on the LT list according to one of the four components of the liver score, including HCC. Inclusion on the list was based on the Milan criteria^5^ or on the 5/5 criteria (5 tumours maximum, with the largest tumour < 5 cm)^31^, before mandatory use of the AFP score from 2013 for all French centres^6^. The AFP score considers three parameters: serum AFP levels, size of the largest tumour, and number of tumours. Patients with a score of ≤ 2 are eligible for enrolment on the transplant list.

If the AFP score is > 2, downstaging treatments may be performed to register or maintain a patient on the list. These mainly include percutaneous ablation, surgical resection or chemoembolization. The decision to perform bridging or downstaging treatments was made after multidisciplinary consultation. Each patient could receive multiple types of treatment.

### LT procedure

In the autotransfusion cohort, all patients underwent orthotopic whole-liver transplantation. The caval anastomosis was created using the piggy back technique or cavacaval side-to-side anastomosis (preservation of the recipient's vena cava)^32^. A temporary portocaval anastomosis was constructed in selected patients depending on intraoperative findings^33^.

### Management of intraoperative blood salvage with autotransfusion and allogeneic transfusion

Intraoperative autotransfusion was enabled by the use of a Cell Saver 5^®^ (Haemonetics, Braintree, MA, USA) with an antileucocyte filter, followed by use of a Cell Saver Elite^®^ (Haemonetics) from 2018. Blood was collected in the operating field via a dedicated suction cannula, after evacuation of any ascites, in a 225-ml bowl with an anticoagulant solution. Once a sufficient volume of blood had been collected, it was washed and centrifuged to form 1 unit of packed RBCs with a haematocrit level between 50 and 60%. The decision regarding intraoperative transfusion was made at the discretion of the anaesthetist in both cohorts. The practice of the anaesthesia team in the autotransfusion cohort was to discharge patients from the operating theatre with a haemoglobin level as close as possible to 10 g/dl. If intraoperative autotransfusion was insufficient, allogeneic transfusion was also used in this cohort.

### Follow-up

Oncological follow-up of patients was performed by thoracoabdominopelvic CT with contrast and serum AFP measurement every 3 months in the absence of intercurrent events. Recurrence and survival information was obtained by consulting the electronic patient file or by telephone call on 1 January 2023, ensuring at least 3 years of follow-up for censored patients.

### Ethics

In accordance with French Ethics Law, patients were informed that their data had been collected and used for this study. Under French Ethical and Regulatory Law, retrospective studies based on the use of routine healthcare data do not have to be submitted to an ethics committee but must be declared to the French Data Protection Authority (CNIL). Personal and medical data were collected and processed electronically. Toulouse University Hospital Centre has signed a commitment to comply with the MR-004 Methodological Guidelines of the CNIL. After evaluation and validation by the Data Protection Officer and in compliance with the General Data Protection Regulation, this study fulfilled all the criteria and is therefore registered in the Toulouse University Hospital Centre's Retrospective Studies Register (registration number RnIPH 2023-41). This study was approved by the Toulouse University Hospital Centre, confirming that all ethical requirements have been fulfilled.

### Statistical analysis

Characteristics of the population are described using standard statistics: quantitative variables by the median (interquartile range, i.q.r.), and qualitative variables by absolute numbers and percentages. The number of patients with missing data was reported for each variable.

Comparisons between groups were made by means of the χ^2^ test or Fisher's exact test for qualitative variables, and the Mann–Whitney *U* test for quantitative variables. OS was defined as the time from the date of transplantation to the date of death or last contact for censored patients. RFS was defined as the time between the date of transplantation and the date of first recurrence or date of death. Patients alive and without recurrence were censored at the date of last contact. Survival rates were estimated using the Kaplan–Meier method with 95% confidence intervals. Univariable and multivariable analyses were undertaken using the log rank test and Cox proportional hazards model. Hazards ratios (HRs) are shown with 95% confidence intervals. The proportional hazards assumption was evaluated using the Schoenfeld residual test. The multivariable analysis included the variable of interest (use of intraoperative autotransfusion) and statistically significant (at level of 5%) and clinically relevant variables from the univariable analysis.

A sensitivity analysis was conducted to evaluate the incidence of recurrence using a competing-risk methodology, considering death as a competing event. The method of Fine and Gray was used, and sub-HRs are reported with corresponding 95% confidence intervals.

A propensity score analysis was undertaken to account for differences in baseline preoperative characteristics in the two cohorts. Patients were matched on propensity scores using the nearest-neighbour method with a caliper (1 : 1 without replacement). Recommendations from the literature were used to calculate a caliper of 5%, taking the standard deviation of the probability of the propensity score, multiplied by 0.2^34^. The propensity scores were defined as the probability of having an intraoperative autotransfusion. This was estimated using a logistic regression model. The following co-variables were included in the model: AFP score at the time of registration (0 or > 0); previous tumour treatment, pretransplant creatinine level, preoperative Child–Pugh grade, time on waiting list, non-alcoholic steatohepatitis (NASH), and hepatitis C virus (HCV) infection. The selection was carried out using backward selection at a significance level of 5%, which excluded the Milan criteria at registration. The number of tumours at registration (412) and downstaging for registration on the list (485) were not retained for the final model in view of the large number of missing data. To evaluate matching balance, standardized mean differences (SMDs) were computed for the co-variables both before and after matching (SMD < 0.1 was considered as negligible imbalance and SMD < 0.2 as acceptable imbalance).

All analyses were performed with Stata^®^ version 16 software (StataCorp, College Station, TX, USA). All tests used were two-sided and *P* < 0.050 was considered statistically significant.

## Results

**Fig. 1:**
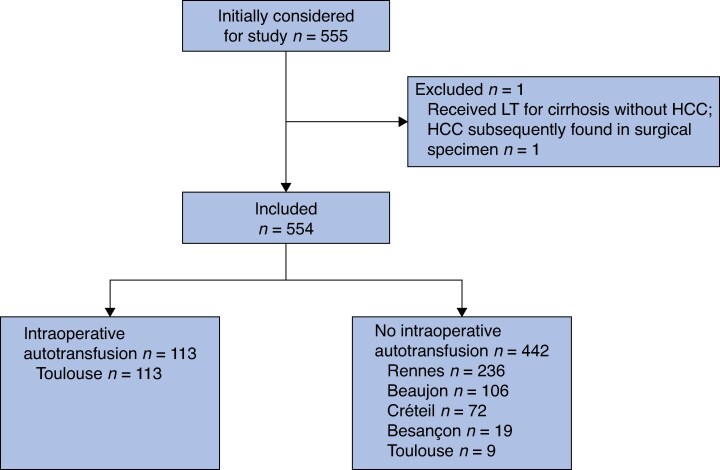
Study flow chart LT, liver transplantation; HCC, hepatocellular carcinoma.

Between 1 January 2011 and 1 January 2020, 124 patients underwent LT for liver cirrhosis complicated by HCC at Toulouse University Hospital Centre. Among these, 113 received an intraoperative autotransfusion and constituted the autotransfusion cohort. The control cohort consisted of 442 patients with cirrhosis complicated by HCC undergoing LT without autotransfusion at the University Hospital Centres of Rennes (236 patients), Beaujon (106), Créteil (72), Besançon (19), and Toulouse (9) between 1 March 2013 and 1 January 2018. One patient who received LT for cirrhosis without HCC, with HCC subsequently found in the surgical specimen, was excluded from the analysis. In total, 554 patients were included in the study (*Fig. 1*).

### Patients undergoing LT for HCC with use of intraoperative blood salvage (autotransfusion cohort)

Preoperative characteristics of the autotransfusion cohort are outlined in *Table 1*. Demographic data reflect the epidemiology of HCC, with a majority of men (88.5%) and a median age at transplantation of 61 (i.q.r. 56–66) years. The three main aetiologies of cirrhosis were alcohol (54.0%), HCV infection (36.3%), and NASH (18.6%). At the time of registration on the LT waiting list, 76.1% of patients met the Milan criteria. The median Model for End-stage Liver Disease score was 12 (i.q.r. 8–16) and the Child–Pugh grade was A or B in most patients. HCC waiting list treatment was undertaken in 89.4% of patients. Only 10.6% of patients were registered after downstaging. AFP scores at the time of registration were 0 in the majority of patients (58.4%), with only four having a score greater than 2 (3.5%). The median waiting time on the transplantation list was 374 (range 2–862; i.q.r. 231–449) days.

**Table 1:** Preoperative data for study and control cohorts

	Total (*n* = 554)	Intraoperative autotransfusion	
		No (*n* = 441)	Yes (*n* = 113)
**Sex**			
Male	501 (90.4%)	401 (90.9%)	100 (88.5%)
Female	53 (9.6%)	40 (9.1%)	13 (11.5%)
Age at time of transplant (years), median (i.q.r.)	61 (56–65)	61 (56–64)	61 (56–66)
BMI (kg/m^2^), median (i.q.r.)	27.0 (24.2–30.4)	27.1 (24.4–30.5)	26.7 (23.7–29.4)
**Pretransplant serum creatinine (mmol/L), median (i.q.r.)**	74 (64–91)	74 (63–90)	79 (66–100)
Missing	7	7	0
MELD score at time of registration, median (i.q.r.)	11 (8–16)	11 (8–16)	12 (8–16)
**Aetiology of cirrhosis**			
Alcohol	332 (59.9%)	271 (61.5%)	61 (54.0%)
HCV	132 (23.8%)	91 (20.6%)	41 (36.3%)
NASH	48 (8.7%)	27 (6.1%)	21 (18.6%)
HBV	33 (6.0%)	23 (5.2%)	10 (8.8%)
**Registration data**			
Milan criteria at time of registration			
No	64 (12.0%)	37 (8.8%)	27 (23.9%)
Yes	469 (88.0%)	383 (91.2%)	86 (76.1%)
Missing	21	21	0
AFP score at time of registration			
0	406 (77.6%)	340 (82.9%)	66 (58.4%)
> 0	117 (22.4%)	70 (17.1%)	47 (41.6%)
1	72 (13.8%)	45 (11.0%)	27 (23.9%)
2	36 (6.9%)	20 (4.9%)	16 (14.2%)
3	5 (1.0%)	3 (0.7%)	2 (1.8%)
4	3 (0.6%)	1 (0.2%)	2 (1.8%)
6	1 (0.2%)	1 (0.2%)	0 (0%)
Missing	31	31	0
Preoperative Child–Pugh grade			
A	317 (57.2%)	264 (59.9%)	53 (46.9%)
B	161 (29.1%)	120 (27.2%)	41 (36.3%)
C	76 (13.7%)	57 (12.9%)	19 (16.8%)
Time on waiting list (days), median (i.q.r.)	325.5 (171–434)	301 (161–431)	374 (231–449)
Missing	72	72	0
Preregistration downstaging			
No	384 (79.2%)	283 (76.1%)	101 (89.4%)
Yes	101 (20.8%)	89 (23.9%)	12 (10.6%)
Missing	69	69	0
Waiting list tumour treatment			
No	262 (48.2%)	250 (58.0%)	12 (10.6%)
Yes	282 (51.8%)	181 (42.0%)	101 (89.4%)
Missing	10	10	0

Values are *n* (%) unless otherwise stated. i.q.r., Interquartile range; BMI, body mass index; MELD, Model for End-stage Liver Disease; HCV, hepatitis C virus; NASH, non-alcoholic steatohepatitis; HBV, hepatitis B virus; AFP, α-fetoprotein.

During surgery, all patients received an autotransfusion with a median volume of 1500 (range 200–15 800; i.q.r. 726–2500) ml transfused blood (*Table 2*). Additional allogeneic transfusions were performed in 86.7% of the patients, with a median of 5 (range 0–20; i.q.r. 3–7) units packed RBCs. A temporary portocaval anastomosis was created in six patients (5.3%). The average duration of operation (mean) was 292 (range 185–563) min.

**Table 2:** Intraoperative and postoperative data

	Total (*n* = 554)	Intraoperative autotransfusion	
		No (*n* = 441)	Yes (*n* = 113)
**Intraoperative data**			
Allogeneic transfusion of packed RBCs			
No	214 (39.3%)	199 (46.2%)	15 (13.3%)
Yes	330 (60.7%)	232 (53.8%)	98 (86.7%)
Missing	10	10	0
No. of units transfused packed RBCs, median (i.q.r.)	2 (0–5)	1 (0–4)	5 (3–7)
Missing	3	2	1
Autotransfusion volume (ml), median (i.q.r.)	–	–	1500 (726–2500)
**Histological data**			
Active tumour on explanted liver			
No	70 (13.2%)	64 (15.4%)	6 (5.3%)
Yes	459 (86.8%)	352 (84.6%)	107 (94.7%)
Missing	25	25	0
No. of tumours, median (i.q.r.)	2 (1–30)	2 (1–30)	3 (1–15)
Missing	5	5	0
Size of largest tumour (cm), median (i.q.r.)	2.0 (1.2–3.0)	2.0 (1.0–3.0)	2.5 (2.0–3.2)
Missing	41	36	5
Satellite nodules			
No	355 (79.1%)	289 (85.0%)	66 (60.6%)
Yes	94 (20.9%)	51 (15.0%)	43 (39.4%)
Missing	105	101	4
Vascular invasion			
No	362 (79.9%)	270 (78.0%)	92 (86.0%)
Yes	91 (20.1%)	76 (22.0%)	15 (14.0%)
Missing	46	95	6

Values are *n* (%) unless otherwise stated. RBC, red blood cell; i.q.r., interquartile range.

On definitive anatomopathological examination of the surgical specimens, a viable tumour was found in 94.7% of patients, with a median of 3 (range 1–15; i.q.r. 2–5) tumours, the largest being 2.5 (range 0.6–10; i.q.r. 2.0–3.2) cm in diameter (*Table 2*). Tumour vascular invasion and satellite nodules were found in 14.0 and 39.4% of patients, respectively.

Median patient follow-up was 83.3 (95% confidence interval (c.i.) 78.2 to 89.3) months. The recurrence rate in this cohort was 15.9% (18 patients), with a median RFS of 121.1 months (95% c.i. 121.1 months to not reached) and a 5-year RFS rate of 66.3 (95% c.i. 56.5 to 74.4)%. At the date of last contact, 44 patients (38.9%) had died.

### Patients undergoing LT without use of intraoperative blood salvage (control cohort)

The proportions in the two cohorts are reported in *Tables 1* and *2*. Before surgery, serum levels of creatinine tended to be lower in the control cohort, and HCV and NASH were less common (20.6 *versus* 36.3% for HCV; 6.1 *versus* 18.6% for NASH). At the time of registration on the waiting list, patients in the control cohort more often met the Milan criteria (91.2 *versus* 76.1%), had an AFP score of 0 (82.9 *versus* 58.4%), and Child–Pugh A grade disease (59.9 *versus* 46.9%). They also more frequently underwent preregistration downstaging (23.9 *versus* 10.6%), experienced fewer waiting list treatments (42.0 *versus* 89.4%), and had a shorter waiting time on the list (median 301 (i.q.r. 161–431) *versus* 374 (231–449) days). Other demographic and registration data did not show a tendency to differ.

During surgery, there were substantially fewer transfusions of allogeneic RBCs in the control cohort (53.8 *versus* 86.7%) and these were in smaller quantities (median 1 (i.q.r. 0–4) *versus* 5 (3–7)). In terms of surgical specimen histology, this cohort had more inactive tumours (15.4 *versus* 5.3%), fewer tumours (median 2 (i.q.r. 1–3) *versus* 3 (2–5)), and fewer satellite nodules (15.0 *versus* 39.4%) (*Table 2*). Other intraoperative and histological criteria did not seem different in terms of proportions.

Median patient follow-up was 84.6 (95% c.i. 82.9 to 87.2) months. The recurrence rate in this cohort was 14.3% (63 patients), median RFS was not reached, and the 5-year RFS rate was 69.7 (95% c.i. 65.1 to 73.8)%. At the date of last contact, 145 patients (32.9%) had died.

### Survival

#### RFS

**Fig. 2:**
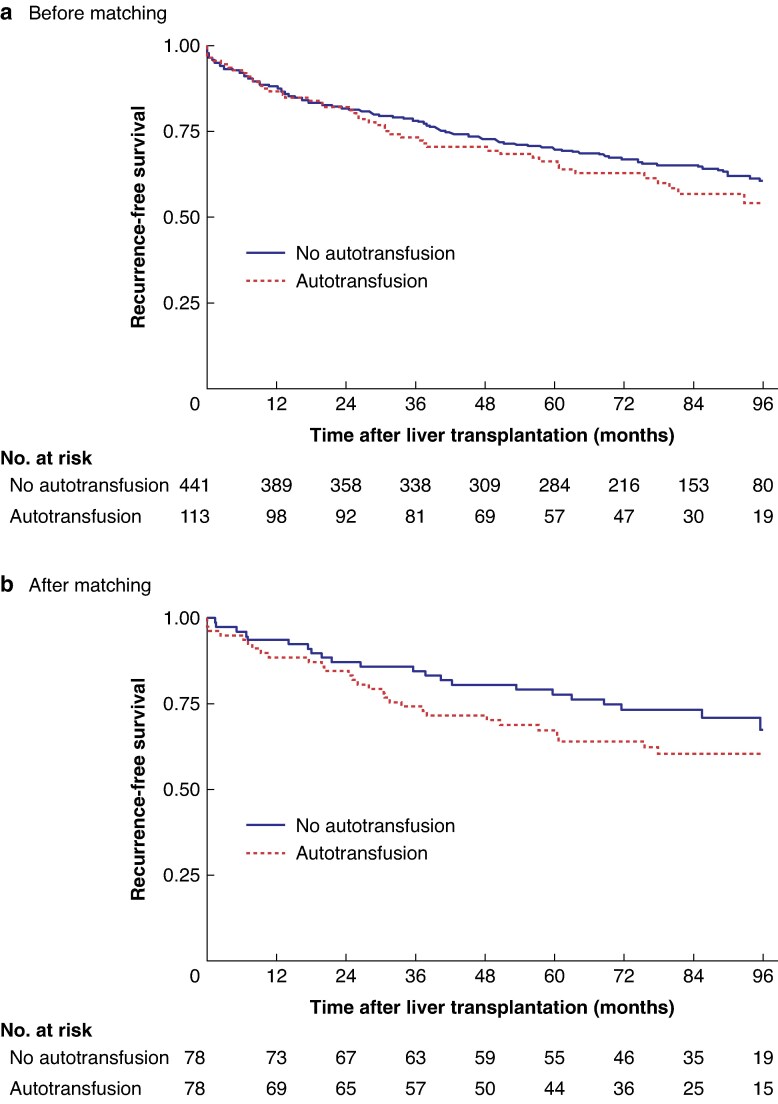
Recurrence-free survival according to the use or non-use of intraoperative autotransfusion **a** Before matching, **b** after matching.

The 5-year RFS rate for the entire population (554 patients) was 69.0 (95% c.i. 64.9 to 72.7)%. Three poor prognostic factors for RFS were found: AFP score > 0 at the time of registration (HR 1.46, 95% c.i. 1.06 to 2.00; *P* = 0.019), vascular invasion on histological analysis (HR 1.77, 1.24 to 2.51; *P* = 0.001), and presence of satellite nodules on histological analysis (HR 1.91, 1.37 to 2.66; *P* < 0.001) (*Table S2*). In contrast, in terms of RFS, the differences observed were not statistically significant with the use of intraoperative autotransfusion both in univariable analysis (5-year RFS 69.7 (65.1 to 73.8)% in control cohort *versus* 66.3 (56.5 to 74.4)% in autotransfusion cohort; HR 1.22, 0.88 to 1.69; *P* = 0.241) (*Fig. 2a*), and multivariable analysis after adjustment for prognostic factors (HR 1.16, 0.78 to 1.73; *P* = 0.465) (*Table S3*). The sensitivity analysis of competing risks, which assessed the risk of recurrence with death considered as a competing event, confirmed this result, and the differences observed were not statistically significant for RFS (sub-HR 1.15, 0.68 to 1.94; *P* = 0.587).

#### OS

**Fig. 3:**
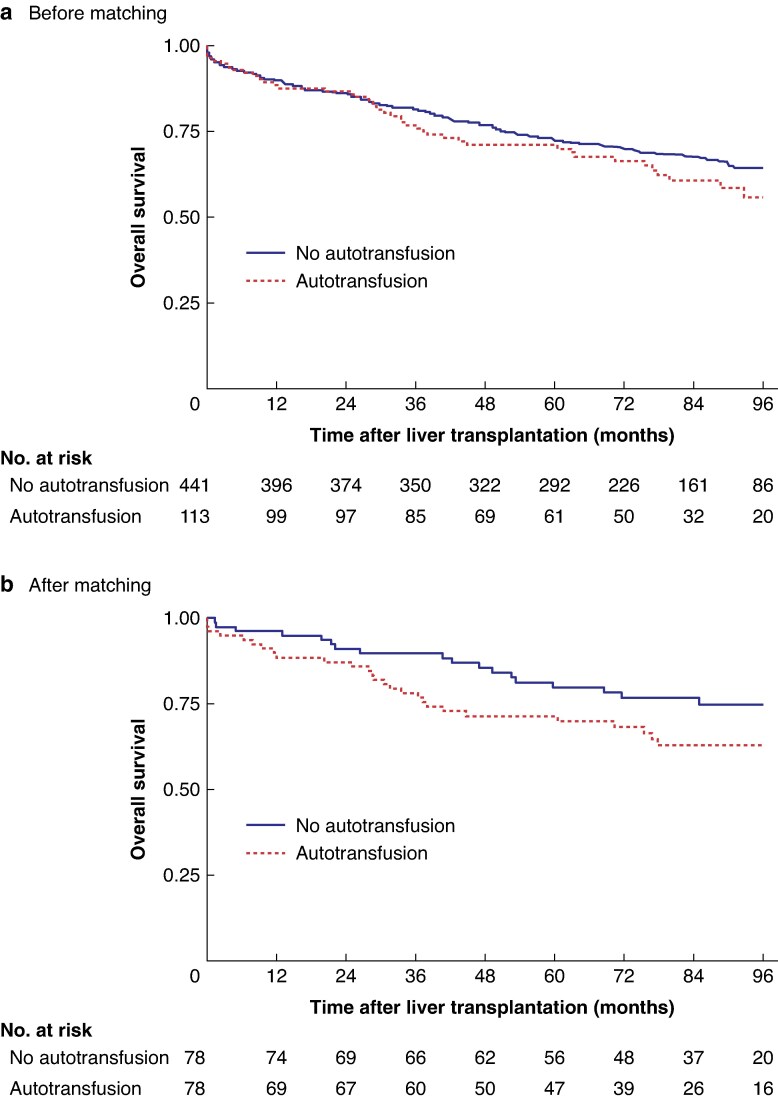
Overall survival according to the use or non-use of intraoperative autotransfusion **a** Before matching, **b** after matching.

The 5-year OS rate for the entire population (554 patients) was 71.9 (95% c.i. 67.9 to 75.5)%. Three poor prognostic factors for OS were found: AFP score > 0 at the time of registration (HR 1.47, 95% c.i. 1.06 to 2.04; *P* = 0.021), vascular invasion (HR 1.79, 1.25 to 2.57; *P* = 0.001), and presence of satellite nodules on histological analysis (HR 1.90, 1.35 to 2.68; *P* < 0.001) (*Table S4*). In contrast, on OS, the differences observed were not statistically significant with the use of intraoperative autotransfusion both in univariable analysis (5-year OS 72.2 (67.7 to 76.2)% in control cohort *versus* 71.1 (61.7 to 78.7)% in autotransfusion cohort; HR 1.22, 0.87 to 1.71; *P* = 0.252) (*Fig. 3a*), and multivariable analysis after adjustment for prognostic factors (HR 1.16, 0.76 to 1.75; *P* = 0.488) (*Table S5*).

### RFS and OS after propensity score matching

Of the 554 patients analysed in the study, 458 had data available for the selected co-variables and were therefore evaluated for the propensity score (*Table S6*). Among these patients, 156 were matched by propensity score: 78 patients in the autotransfusion cohort and 78 in the control cohort. The SMDs used to assess the balance of co-variables before and after matching are presented in *Fig. S1* and *S2* for the preoperative characteristics used for propensity score matching. Main preoperative characteristics were well balanced after matching, but some minor imbalances remained.

#### RFS after matching

After matching, the difference in RFS between the two cohorts was not statistically significant (HR 1.47, 95% c.i. 0.84 to 2.55; *P* = 0.174). The RFS rate at 5 years was 67.1 (95% c.i. 55.3 to 76.5)% in the propensity score-matched autotransfusion cohort *versus* 77.6 (66.5 to 85.5)% in the propensity score-matched control cohort (*Fig. 2b*).

The sensitivity analysis of competing risks showed that the use of intraoperative autotransfusion did not imply a significant difference in risk of recurrence in the matched population (sub-HR 0.92, 0.41 to 2.08; *P* = 0.810).

#### OS after matching

After matching, the difference in OS between the two cohorts was not statistically significant (HR 1.58, 95% c.i. 0.88 to 2.85; *P* = 0.123). The OS rate at 5 years was 71.4 (95% c.i. 59.9 to 80.2)% in the propensity score-matched autotransfusion cohort *versus* 79.8 (68.7 to 87.3)% in the propensity score-matched control cohort (*Fig. 3b*).

## Discussion

This study showed no statistically significant difference in recurrence or mortality with the use of autotransfusion during LT for HCC. Indeed, when intraoperative autotransfusion was used, the difference observed was not statistically significant for the primary endpoint of RFS either before (HR 1.22, 95% c.i. 0.88 to 1.69) or after (HR 1.47, 0.84 to 2.55) propensity score matching, with a 5-year RFS of 67.1 *versus* 77.6%. These results are consistent with previous literature, where the 5-year OS rate in the autotransfusion groups varied from 59.5 to 83% at 5 years^20,25^, with a 5-year RFS from 64 to 86%^18,35^.

These results confirm the absence of a statistically significant difference in RFS and OS with the use of the intraoperative autotransfusion during LT for HCC. This is in line with findings for various types of oncological procedures, including radical prostatectomies^36^, hepatectomies, and pancreatectomies for cancer^37^. With regard to LT for HCC, 11 studies have looked at this procedure (*Table S7*), with autotransfusion group populations ranging from 10 to 283 patients, although most often comprising fewer than 100 patients^16,18–21,24–26,35,38,39^. Notably, a recent meta-analysis^27^ concluded that use of intraoperative blood salvage with autotransfusion in LT for HCC has no influence on the oncological outcomes of patients and reduces the consumption of allogeneic RBCs. However, despite all these results, the routine use of intraoperative blood salvage with autotransfusion is not standard practice and remains highly debated.

In the present analysis, the median volume of autotransfused blood was 1500 ml per transplantation, equivalent to approximately 4–5 units of packed RBCs. This result is consistent with those of similar studies^16,18–21,24–26,35,38,39^, which reported median volumes of autotransfused blood ranging from 750 to 1699 ml. Saving allogeneic packed RBCs is a public health, medicoeconomic, and individual issue. France has experienced a decline in blood donation, with stocks of packed RBCs below the alert threshold for more than half the year^41,42^. At the individual level, it has been well demonstrated that the number of units of allogeneic packed RBCs transfused is associated with incidence of postoperative complications and decreased postoperative survival^40^. In a meta-analysis of colorectal cancer surgery, Acheson *et al.*^11^ found that autologous blood transfusions were associated with a significantly higher rate of postoperative infections, reinterventions, and mortality, as well as longer hospital stay. Furthermore, Wu *et al.*^40^ showed that mortality risk was directly related to transfusion volumes in a cohort of over 4000 patients with colorectal cancer (HR 1.58 for ≤ 4 units packed RBCs; HR 2.32 for > 4 units packed RBCs transfused). This led the European Society of Anaesthesiology and Intensive Care^43^ to issue a strong recommendation in 2022 for the use of intraoperative autotransfusion systems during non-cardiac operations with expected blood loss of > 500 ml.

In the case of cancer surgery, two studies^29,44^ have looked at the presence of circulating tumour cells. In these two studies, no residual tumour cells were found after filtration by this process. No study has specifically looked at the search for circulating tumour cells in patients having LT for HCC. There is currently no solid evidence for the presence of tumour cells after filtration by intraoperative autotransfusion machines.

This research has the limitations inherent to retrospective studies. The autotransfusion cohort was not comparable to the control cohort, particularly as regards the management of tumours on waiting lists, but also waiting times, which are dependent on local expertise. For the authors’ part, they have held a multidisciplinary consultation meeting dedicated to HCC for over 10 years^45^, enabling them to adopt an aggressive treatment strategy including extension of the Milan criteria up to 5^31^, and mandatory use of the AFP score in France.

In addition, a limited number of patients could be included in the propensity score analysis, owing to the limited number of patients who had undergone intraoperative autotransfusion and the large initial difference between the two groups. The methods used did not fully correct for the pre-existing imbalances between the two groups. Finally, the possibility cannot be ruled out that the absence of a statistical difference was due to a lack of power.

Another limitation is that, despite the use of blood salvage with autotransfusion, 86.7% of patients in the autotransfusion cohort received additional autologous blood transfusions, with a median of 5 units packed RBCs per patient. In contrast, fewer patients in the control cohort received transfusions; only 53.8% of these patients received autologous blood transfusions, and a median of 1 unit packed RBCs was transfused per patient. This difference could be explained by variation in the haemoglobin targets on exit from the operating theatre between different anaesthesia teams, as well as differences in fluid management strategies. Overall, the authors’ transfusion practices seem to be in line with those in the majority of international studies, where the amount transfused ranges from 7 to 10 units packed RBCs^8,46,47^ during the perioperative phase (before, during, and after operation).

Finally, information on intraoperative blood loss, details of intraoperative technique, and data on postoperative complications were not available for the control cohort and could not be included in the analysis.

Management of intraoperative bleeding is a critical issue during LT, affecting not only the patients, but also the broader community owing to the limited availability of allogeneic blood products. In this context, the debate around the potential use of intraoperative blood salvage with autotransfusion appears of crucial importance. The present results suggest that intraoperative blood recovery with autotransfusion could be an interesting solution in LT for HCC. However, a study with more patients would allow the possibility to be ruled out that the absence of statistical differences observed here related to a lack of power. A large-scale prospective study seems necessary to validate these results.

## Supplementary Material

zraf101_Supplementary_Data

## Data Availability

The data sets generated and/or analysed during the present study are available from the corresponding author on reasonable request.
