# Supplementary material for: Effect of intraoperative autotransfusion use during liver transplantation for hepatocellular carcinoma on recurrence-free survival: comparative study with propensity score matching
Source: BJS Open. 2025 Sep 9;9(5):zraf101. doi: 10.1093/bjsopen/zraf101 (PMC12419531; doi:10.1093/bjsopen/zraf101)
Supplement: zraf101_Supplementary_Data [file zraf101_supplementary_data.docx]

**Effect of Intraoperative Autotransfusion Use During Liver Transplantations for Hepatocellular Carcinoma on Recurrence-Free Survival: Comparative Study with Propensity Score Matching**

**Authors:** Paul Boulard^1^ MD, Charlotte Maulat^1^ MD, Ana Cavillon^2^, Fabien Robin^3^ MD, Frederica Dondero^4^ MD, Chady Salloum^5^ MD, Celia Turco^6^ MD, Flavy Breheret^3^, Valérie Paradis^5^ MD, PhD, Chetana Lim^5^ MD, Bruno Heyd^6^ MD, PhD, Emmanuel Cuellar^1^ MD, Bertrand Suc^1^ MD, PhD, Daniel Azoulay^5^ MD, PhD, Isabelle Migueres^7^ MD, François Cauchy^4^ MD, PhD, Fabrice Muscari^1^ MD, PhD

**Affiliations:**

1- Digestive Surgery and Transplantation Department, Toulouse University Hospital Center, Toulouse, France

2- Biostatistics & Health Data Science Unit, Claudius-Regaud Institute, IUCT-Oncopole Toulouse, France

3- Rennes University Hospital Center

4- Beaujon University Hospital Center

5- Créteil University Hospital Center

6- Besançon University Hospital Center

1. Anesthesiology Department Toulouse University Hospital Center

**Corresponding Authors:**

Pr Fabrice Muscari^1^, MD, Digestive Surgery and Liver Transplantation Department, Toulouse University Hospital Center, 1 avenue Jean Poulhès, 31059 Toulouse, France. Telephone: +33561322741; Fax: +33561322936; Email: muscari.f@chu-toulouse.fr

**Supplementary Materials - Index**

| **Supplementary Appendixes** |  |
| --- | --- |
| AFP score | *page 2* |
| **Supplementary Figures and Tables** |  |
| **Supplementary Table 1** | *page 3* |
| **Supplementary Table 2** | *page 4* |
| **Supplementary Table 3** | *page 5* |
| **Supplementary Table 4** | *page 6* |
| **Supplementary Table 5** | *page 7* |
| **Supplementary Table 6** | *page 8* |
| **Supplementary Table 7** | *page 9* |
| **Supplementary Figure 1** | *page 10* |
| **Supplementary Figure 2** | *page 10* |
|  |  |

**Supplementary Appendixes**

**Supplementary Appendix 1 – AFP score**

|  | **Points** |
| --- | --- |
| **Maximum Diameter (cm)**  ≤3  3-6  >6 | 0  1  4 |
| **Number of tumours**  1-3  ≥4 | 0  2 |
| **Serum AFP level (μg/L)**  <100  100-1000  >1000 | 0  2  3 |

**Supplementary Figures and Tables**

**Supplementary Table 1** – Data Collection

|  | Study Cohort | Control Cohort |
| --- | --- | --- |
| Preoperative Data |  |  |
| Gender - Age - BMI* - Dialysis  Creatinine - INR - Anticoagulants - Etiology of cirrhosis - Registration date - Waiting time - MELD - AFP - AFP Score - Child Pugh - Number and size of tumors - Milan Criteria - Waiting list treatment - Downstaging | + | + |
| Intraoperative Data |  |  |
| Autotransfused blood volume - Operating time - Temporary PCA | + | - |
| Allogeneic transfusions - Number of units of packed RBCs | + | + |
| Postoperative Data |  |  |
| Clavien-Dindo score ≥ 3 - Further surgery within the first 90 days - Death within the first 90 days - Length of hospitalization in the Intensive Care Unit - Length of hospital stay. | + | - |
| Histological Data |  |  |
| Number and size of tumors - Tumor on explanted liver - Satellite nodules - Vascular invasion - | + | + |
| Follow-Up Data |  |  |
| Date of last news - Living or deceased status - Oncological recurrence - Location of recurrence - Recurrence-free survival - Overall survival. | + | + |

AFP Score: Alpha-Foeto-Protein Score ; AFP: Alpha-Foeto-Protein ; BMI : Body Mass Index ; INR: International Normalized Ratio ; MELD: Model for End stage Liver Disease ; PCA: Porto-Caval Anastomosis ; RBC: Red Blood Cell Concentrates

**Supplementary Table 2** - Factors associated with Recurrence-Free Survival in the whole patient population– Univariable Analysis (n=554)

|  | Event/Number | S(t=60 months) | HR (95%CI) | p-value |
| --- | --- | --- | --- | --- |
| **Intraoperative Autotransfusion**  No  Yes | 156/441  47/113 | 69.7 [65.1 – 73.8]  66.3 [56.5 – 74.4] | 1.0  1.2 [0.9 – 1.7] | 0.241 |
| **Sex**  Male  Female | 183/501  20/53 | 69.4 [65.1 – 73.3]  64.7 [52.8 – 79.0] | 1.0  1.1 [0.7 – 1.7] | 0.823 |
| **Age at transplantation**  < 60 years  ≥ 60 years | 89/244  114/310 | 70.0 [63.7 – 75.4]  68.2 [62.6 – 73.1] | 1.0  1.1 [0.79 – 1.38] | 0.740 |
| **Milan Criteria at the Time of Registration**  No  Yes | 29/64  166/469 | 61.6 [48.4 – 72.4]  70.1 [65.6 – 74.0] | 1.0  0.8 [0.5 – 1.1] | 0.162 |
| **Preregistration Downstaging**  No  Yes | 136/384  30/101 | 69.9 [65.0 – 74.2]  74.3 [64.3 – 81.9] | 1.0  0.8 [0.5 – 1.2] | 0.131 |
| **Waiting Time on List (Days)** | 167/482 |  | 1.0 [1.0 – 1.0] | 0.934 |
| **Preoperative Child Pugh**  A  B  C | 110/317  62/161  31/76 | 70.6 [65.2 – 75.3]  68.2 [60.3 – 74.9]  63.5 [51.4 – 73.4] | 1.0  1.2 [0.9 – 1.6]  1.2 [0.8 – 1.8] | 0.470 |
| **Previous Tumor Treatment**  No  Yes | 94/262  106/282 | 67.2 [61.1 – 72.6]  70.2 [64.4 – 75.3] | 1.0  1.0 [0.8 – 1.3] | 0.984 |
| **AFP Score at the Time of Registration**  = 0  > 0 | 136/406  53/117 | 72.3 [67.6 – 76.4]  59.2 [49.5 – 67.7] | 1.0  1.4 [1.1 – 2.0] | **0.019** |
| **Active tumor on the explant**  No  Yes | 21/70  175/459 | 76.6 [64.6 – 85.0]  67.4 [62.9 – 71.5] | 1.0  1.4 [0.89 – 2.2] | 0.14 |
|  |  |  |  |  |
| **Vascular Invasion**  No  Yes | 118/362  43/91 | 74.1 [69.2 – 78.3]  59.6 [48.7 – 69.0] | 1.0  1.8 [1.2 – 2.5] | **0.001** |
| **Satellite Nodules**  No  Yes | 110/355  51/94 | 74.7 [69.8 – 79.0]  55.9 [45.2 – 65.3] | 1.0  1.9 [1.4 – 2.7] | **<0.001** |

AFP Score: Alpha-Foeto-Protein Score

**Supplementary Table 3: Recurrence Free Survival Multivariable analysis**

|  | **HR [95%CI]** | **p-value** |
| --- | --- | --- |
| **Intraoperative Autotransfusion** |  |  |
| No | 1.00 |  |
| Yes | 1.16 [0.78; 1.73] | 0.465 |
|  |  |  |
| **AFP Score at the Time of Registration** |  |  |
| 0 | 1.00 |  |
| >0 | 1.30 [0.89; 1.90] | 0.178 |
|  |  |  |
| **Vascular Invasion** |  |  |
| No | 1.00 |  |
| Yes | 1.50 [1.01; 2.22] | **0.046** |
|  |  |  |
| **Satellite Nodules** |  |  |
| No | 1.00 |  |
| Yes | 1.45 [0.98; 2.14] | *0.062* |

**Supplementary Table 4:** Prognostic Factors for Overall Survival - Univariable Analysis

|  | Event/Number | S(t=60 months) | HR [95%CI] | p-value |
| --- | --- | --- | --- | --- |
| **Intraoperative Autotransfusion**  No  Yes | 145/441  44/113 | 72.2 [67.7 – 76.2]  71.1 [61.7 – 78.7] | 1.0  1.2 [0.9 – 1.7] | 0.252 |
| **Sex**  Male  Female | 171/501  18/53 | 72.3 [68.1 – 76.0]  67.8 [52.8 – 79.0] | 1.0  1.0 [0.6 – 1.6] | 0.582 |
| **Age at transplantation**  < 60 years  ≥ 60 years | 82/244  107/310 | 73.6 [67.5 – 78.7]  70.6 [65.0 – 75.4] | 1.0  1.1 [0.8 – 1.4] | 0.58 |
| **Milan Criteria at the Time of Registration**  No  Yes | 28/64  153/469 | 69.7 [56.7 – 79.5]  72.5 [68.1 – 76.3] | 1.0  0.7 [0.5 – 1.1] | 0.109 |
| **Preregistration Downstaging**  No  Yes | 128/384  27/101 | 72.1 [67.3 – 76.4]  77.2 [67.4 – 84.4] | 1.0  0.7 [0.5 – 1.1] | 0.131 |
| **Waiting Time on List (days)** | 155/482 |  | 1.0 [1.0 – 1.0] | 0.985 |
| **Preoperative Child Pugh**  A  B  C | 103/317  57/161  29/76 | 73.6 [68.4 – 78.2]  70.6 [62.7 – 77.1]  67.1 [55.0 – 76.7] | 1.0  1.2 [0.8 – 1.6]  1.2 [0.8 – 1.9] | 0.535 |
| **Previous Tumor Treatment**  No  Yes | 87/262  99/282 | 70.1 [64.1 – 75.3]  73.2 [67.5 – 78.1] | 1.0  1.0 [0.8 – 1.3] | 0.995 |
| **AFP Score at the Time of Registration**  = 0  > 0 | 125/406  50/117 | 74.4 [69.8 – 78.4]  65.9 [56.2 – 73.9] | 1.0  1.5 [1.1 – 2.0] | **0.021** |
| **Active tumor on the explant**  No  Yes | 19/70  163/459 | 78.0 [66.1 – 86.1]  70.7 [66.3 – 74.7] | 1.0  1.4 [0.9 – 2.3] | 0.13 |
| **Vascular Invasion**  No  Yes | 108/362  41/91 | 77.2 [72.4 – 81.2]  62.2 [51.1 – 71.5] | 1.0  1.8 [1.3 – 2.6] | **0.001** |
| **Satellite Nodules**  No  Yes | 100/355  49/94 | 77.2 [72.4 – 81.3]  61.3 [50.6 – 70.4] | 1.0  1.9 [1.4 – 2.7] | **<0.001** |

AFP Score: Alpha-Foeto-Protein Score

**Supplementary Table 5: Overall Survival Multivariable analysis**

|  | **HR [95%CI]** | **p-value** |
| --- | --- | --- |
| **Intraoperative Autotransfusion** |  |  |
| No | 1.00 |  |
| Yes | 1.16 [0.76; 1.75] | 0.488 |
|  |  |  |
| **AFP Score at the Time of Registration** |  |  |
| 0 | 1.00 |  |
| >0 | 1.33 [0.89; 1.97] | 0.159 |
|  |  |  |
| **Vascular Invasion** |  |  |
| No | 1.00 |  |
| Yes | 1.52 [1.01; 2.29] | 0.047 |
|  |  |  |
| **Satellite Nodules** |  |  |
| No | 1.00 |  |
| Yes | 1.44 [0.96; 2.16] | ***0.076*** |

**Supplementary Table 6 :** Characteristics of the propensity score matched cohort

|  |  | Intraoperative Autotransfusion | | p value |
| --- | --- | --- | --- | --- |
|  | Total | No | Yes |  |
|  | (n = 156) | (n = 78) | (n = 78) |  |
| **AFP Score at the time of registration**  = 0  > 0 | 117 (75.0)  39 (25.0) | 60 (76.9)  18 (23.1) | 57 (73.1)  21 (26.9) | 0.58 |
| **Pretransplant creatinine serum levels*** | 74.0 (43.0 – 312.0) | 73.0 (44.0 - 312.0) | 77.0 (43.0 - 199.0) | 0.64 |
| **Preoperative Child Pugh**  A  B  C | 84 (53.8)  48 (30.8)  24 (15.4) | 41 (52.6)  24 (30.8)  13 (16.7) | 43 (55.1)  24 (30.8)  11 (14.1) | 0.90 |
| **Previous tumor treatment**  No  Yes | 23 (14.7)  133 (85.3) | 11 (14.1)  67 (85.9) | 12 (15.4)  66 (84.6) | 0.82 |
| **Waiting time on list (days)**  Median  (Range) | 358.5  (1.0 – 1167.0) | 347.5  (1.0 – 1167.0) | 365.5  (2.0 – 862.0) | 0.69 |
| **NASH**  No  Yes | 139 (89.1)  17 (10.9) | 68 (87.2)  10 (12.8) | 71 (91.0)  7 (9.0) | 0.44 |
| **HCV**  No  Yes | 94 (60.3)  62 (39.7) | 47 (60.3)  31 (39.7) | 47 (60.3)  31 (39.7) | 1.00 |

* IQR

**Supplementary Table 7 :**

| **Reference** | **Year of Publication** | **Design** | **Control Adjustment** | **Autotransfusion Volume (ml)** | **Population** | | **OS at 5 years** | ***p-value*** | **RFS at 5 years** | ***p-value*** | **Recurrence** | ***p-value*** |
| --- | --- | --- | --- | --- | --- | --- | --- | --- | --- | --- | --- | --- |
| Akbulut et al.^20^ | 2013 | Retrospective  Monocentric | None | - | **Total** | **83** |  |  |  |  |  |  |
|  |  |  |  |  | BS+ | 24 | - |  | - |  | 29.2 | *0.7* |
|  |  |  |  |  | BS- | 59 | - |  | - |  | 25.4 |  |
| Araujo et al.^21^ | 2016 | Retrospective  Monocentric | None | - | **Total** | **158** |  |  |  |  |  |  |
|  |  |  |  |  | BS+ | 122 | 59.5 | *0.237* | 85.0 | *0.953* | - |  |
|  |  |  |  |  | BS- | 36 | 64.5 |  | 78.8 |  | - |  |
| Foltys et al.^19^ | 2011 | Retrospective  Monocentric | None | 1130 | **Total** | **126** |  |  |  |  |  |  |
|  |  |  |  |  | BS+ | 40 | 60.0 | *0.67* | 86.0 | *0.29* | - |  |
|  |  |  |  |  | BS- | 96 | 57.0 |  | 68.0 |  | - |  |
| Han et al.^22^ | 2016 | Retrospective  Monocentric | None | 1391 | **Total** | **397** |  |  |  |  |  |  |
|  |  |  |  |  | BS+ | 283 | - |  | - |  | 20.3 | *0.579* |
|  |  |  |  |  | BS- | 114 | - |  | - |  | 24.1 |  |
| Ivanics et al.^26^ | 2021 | Retrospective  Monocentric | PS | 750 | **Total** | **110** |  |  |  |  |  |  |
|  |  |  |  |  | BS+ | 76 | 83.0 | *0.79* | - |  | 1.8 | *0.55* |
|  |  |  |  |  | BS- | 34 | 87.8 |  | - |  | 3.2 |  |
| Kim et al.^40^ | 2013 | Retrospective  Monocentric | None | 1590 | **Total** | **230** |  |  |  |  |  |  |
|  |  |  |  |  | BS+ | 121 | - |  | 83.3 | *0.314* | - |  |
|  |  |  |  |  | BS- | 109 | - |  | 77.4 |  | - |  |
| Kwon et al.^27^ | 2021 | Retrospective  Monocentric | None | 811 | **Total** | **349** |  |  |  |  |  |  |
|  |  |  |  |  | BS+ | 220 | 78.8 | *0.103* | 81.5 | *0.59* | - |  |
|  |  |  |  |  | BS- | 129 | 66.4 |  | 70.5 |  | - |  |
| Muscari et al.^17^ | 2005 | Retrospective  Monocentric | None | 1558 | **Total** | **47** |  |  |  |  |  |  |
|  |  |  |  |  | BS+ | 31 | - |  | - |  | 6.4 | *0.9* |
|  |  |  |  |  | BS- | 16 | - |  | - |  | 6.3 |  |
| Nutu et al.^36^ | 2021 | Retrospective  Monocentric | PS | 1014 | **Total** | **378** |  |  |  |  |  |  |
|  |  |  |  |  | BS+ | 192 | - |  | 64.0 | *0.355* | - |  |
|  |  |  |  |  | BS- | 186 | - |  | 68.0 |  | - |  |
| Pinto et al.^25^ | 2021 | Retrospective  Monocentric | None | - | **Total** | **156** |  |  |  |  |  |  |
|  |  |  |  |  | BS+ | 122 | 67.7 | *0.77* | 66.5 | *0.74* | 10.6 | *0.76* |
|  |  |  |  |  | BS- | 34 | 67.5 |  | 64.1 |  | 8.8 |  |
| Weller et al.^41^ | 2021 | Retrospective  Monocentric | None | 1699 | **Total** | **51** |  |  |  |  | 19.6 |  |
|  |  |  |  |  | BS+ | 39 | - |  | - |  | - |  |
|  |  |  |  |  | BS- | 12 | - |  | - |  | - |  |
| Our study | 2023 | Retrospective  Multicentric | PS | 1500 | **Total** | **554** |  |  |  |  |  |  |
|  |  |  |  |  | BS+ | 113 | 71.1 | *0.252* | 68.0 | *0.505* | 14.3 | *NS* |
|  |  |  |  |  | BS- | 441 | 72.2 |  | 74.0 |  | 15.9 |  |

**Supplementary Figure 1** – Standardized Mean Difference before and after matching


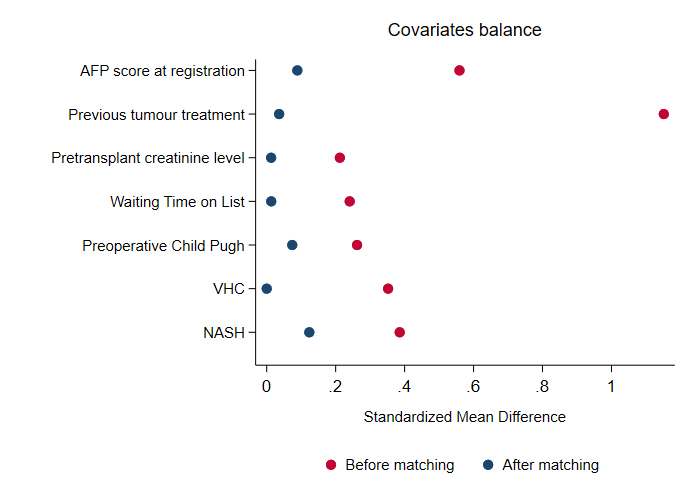


**Supplementary Figure 2** – Histograms displaying the distribution of propensity scores before and after matching
